# Rheotaxis of Larval Zebrafish: Behavioral Study of a Multi-Sensory Process

**DOI:** 10.3389/fnsys.2016.00014

**Published:** 2016-02-23

**Authors:** Raphaël Olive, Sébastien Wolf, Alexis Dubreuil, Volker Bormuth, Georges Debrégeas, Raphaël Candelier

**Affiliations:** Laboratoire Jean Perrin, Université Pierre et Marie Curie, Sorbonne Universités, Centre National de la Recherche Scientifique 8237Paris, France

**Keywords:** zebrafish, rheotaxis, behavior, vision, lateral line, multi-sensory integration

## Abstract

Awake animals unceasingly perceive sensory inputs with great variability of nature and intensity, and understanding how the nervous system manages this continuous flow of diverse information to get a coherent representation of the environment is arguably a central question in systems neuroscience. Rheotaxis, the ability shared by most aquatic species to orient toward a current and swim to hold position, is an innate and robust multi-sensory behavior that is known to involve the lateral line and visual systems. To facilitate the neuroethological study of rheotaxic behavior in larval zebrafish we developed an assay for freely swimming larvae that allows for high experimental throughtput, large statistic and a fine description of the behavior. We show that there exist a clear transition from exploration to counterflow swim, and by changing the sensory modalities accessible to the fishes (visual only, lateral line only or both) and comparing the swim patterns at different ages we were able to detect and characterize two different mechanisms for position holding, one mediated by the lateral line and one mediated by the visual system. We also found that when both sensory modalities are accessible the visual system overshadows the lateral line, suggesting that at the larval stage the sensory inputs are not merged to finely tune the behavior but that redundant information pathways may be used as functional fallbacks.

## 1. Introduction

Positive rheotaxis is the innate behavior shared by most fishes and amphibians to turn to face into an oncoming current, and hold position with minimum expenditure of energy (Arnold and Weihs, 1977). Its widespread nature and the fact that it imposes to manage many sensory cues of different nature (visual, vestibular, hydromechanical, proprioceptive) that are available to the animal makes the generic neuronal processes at play of great interest from a fundamental perspective.

The term *rheotaxis* actually encompasses two distinct behavioral phases, namely orientation of the body along stream lines and counterflow swim to hold position. Position holding is often considered to rely on the sole visual system and in particular on the so-called optomotor reflex (Orger et al., 2000, 2008; Portugues and Engert, 2009). Regarding body orientation, it has been considered for a long time that it could not originate from hydromechanical cues based on the false assumption that when a fish is carried away in a stream there is no relative motion between the fish and its surrounding medium (Lyon, 1904; Arnold, 1974). In real situations there are always velocity gradients created by walls, obstacles, sheared flows, or turbulence which can be captured by the lateral line, and it has been shown that superficial neuromasts mediate the body reorientation behavior (Montgomery et al., 1997) even in the absence of visual cues (Baker and Montgomery, 1999). Most subsequent studies then considered that the lateral line is the dominant sensory system in controlling body orientation (Chagnaud et al., 2007; Olszewski et al., 2012; Suli et al., 2012).

This categorization—the lateral line mediates body orientation and the optomotor response mediates counterflow swim—is however too simplistic to explain the rich behavior of aquatic species. For instance there are evidences that visual (Suli et al., 2012) or olfactory (Baker and Montgomery, 1999) cues help fishes to orient themselves and that some fishes deprived of both visual and hydomechanical cues can still perform rheotaxis (Van Trump and McHenry, 2013), supposedly by means of tactile perception. The whole rheotaxis process should thus be regarded as a multisensory task (Bak-Coleman and Coombs, 2014) and it remains to elucidate, for an animal that is able to perform body orientation and position holding when deprived of any of the lateral line or the visual system, what are the relative contributions of each sensory input during each of the two rheotaxic phases. For each phase, two main hypotheses can be drawn up: (i) there is a dominant modality that inhibits the inputs from the other modality or (ii) there is a weighted integration of both inputs, such as Bayesian inference (Pouget et al., 2013) for instance, that would result in a intermediary behavior.

Here, we investigate the rheotaxic behavior of freely-swimming larval zebrafish in a radial flow assay. This vertebrate model presents a number of advantages for studying the neural basis of behavior (Portugues and Engert, 2009) and, in particular, its translucency and genetic tractability allow for non-invasive imaging of brain-wide neural activity (Orger et al., 2008; Ahrens et al., 2013; Panier et al., 2013). We tracked thousands of larvae in different sensory conditions (with or without light, with or without hydomechanical cues) and categorized the discrete bouts in sequences of exploration swim and counterflow swim. The counterflow swim sequences (CSS) showed clear position holding, both angularly and radially, when visual or hydromechanical cues could be exploited. We observed that the position holding mechanism is different when the larva has only access to visual or hydromechanical cues and that, except for the CSS initiation where the lateral line system allows for earlier triggering, the visual system is the dominant modality in shaping the swimming patterns during CSS.

## 2. Results

### 2.1. Rheotaxis assay

**Figure 1:**
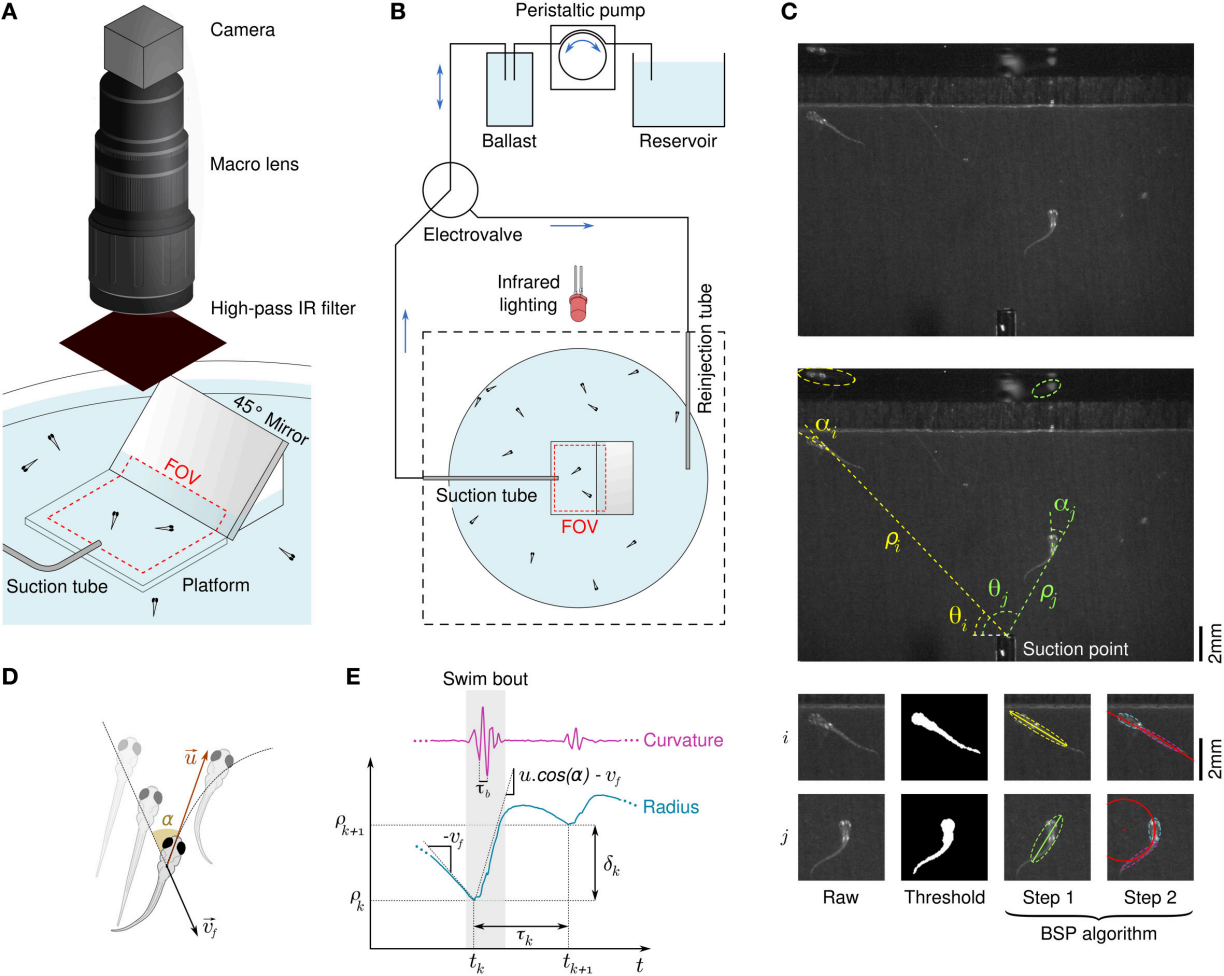
**Behavioral response of freely swimming zebrafish larvae to a radial flow**. **(A)** Schematic diagram of the rheotaxis assay. Zebrafish larvae are spread all across the pool and a thin tube creates a radial flow by aspiration. The field of view (FOV) is a rectangle in the center of the assay which comprises a platform that locally raises the ground and the bottom part of a 45° mirror. An infrared-sensitive camera continuously images the FOV at a framerate of 250 Hz. **(B)** Schematic view of the flow control system. The suction tube is connected to a ballast, a peristaltic pump, and a reservoir. A computer-driven electrovalve allows to reinject water orthoradially to periodically randomize the larvae positions. **(C)** Illustration of the image processing algorithm. Raw grayscale images (top) are substracted to a background image and thresholded to materialize binarized larvae bodies (bottom left), from which polar coordinates [ρ(*t*), θ(*t*)] are derived (center). A BSP tree (bottom-right) is used to obtain the equivalent ellipses of the head and tail and define the body angle (head angle) α(*t*) and body curvature κ(*t*). **(D)** Scheme defining the fluid velocity $\vec{v_{f}}$ and the bout impulse speed $\vec{u}$. **(E)** Swim bouts are located on the basis of the curvature's trace. For each swim bout *k* we define the radius ρ_*k*_ where the bout started and the inter-bout delay / distance, respectively τ_*k*_ = *t*_*k* + 1_ − *t*_*k*_ and δ_*k*_ = ρ_*k* + 1_ − ρ_*k*_.

To study rheotaxis in freely swimming larval zebrafish we used the radial flow geometry proposed in Olszewski et al. (2012) in an assay allowing for high experimental throughtput. Clutches of tenths of wild-type larvae from 5 to 9 days post fertilization (dpf) were placed in the assay and series of aspiration from a suction point followed by water reinjection were applied (Figures 1A,B). Among the dozens of larvae present in the device, only the few lying in the field of view at the center of the assay were imaged during the suction phase by a high speed camera at 250 Hz. Flow rates ranging over a decade were applied with a cycle-to-cycle randomization. The reinjection flow randomly reset the positions of the larvae in the assay at each cycle, ensuring a renewal of the population lying in the field of view. We used an *a posteriori* image processing method to automatically track the larvae, extract body curvatures, and define swim bouts with great accuracy (see Materials and Methods, Figure 1C and Supplementary Video 1). The notation of quantities relative to swim bouts are introduced in Figures 1D,E.

**Figure 2:**
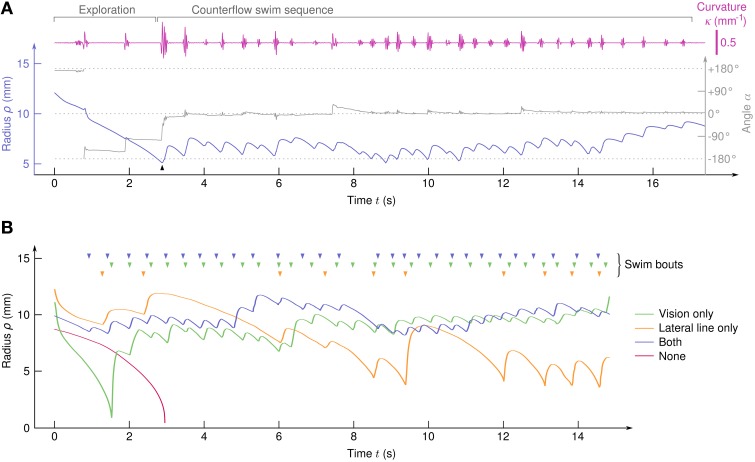
**Typical trajectories**. **(A)** Traces of ρ(*t*), α(*t*), and κ(*t*) for a sample trajectory of a larva with both the visual system and the lateral line. A counterflow swim sequence of 31 bouts starts at *t*≃3*s* (arrow). **(B)** Typical trajectories of four larvae in the different sensory conditions. (top) Arrowheads indicate the swim bouts.

The larvae showed robust positive rheotaxis, i.e., alignment toward the flow and counterflow swimming in the form of sequences of bouts (Figure 2A). With 52 clutches submitted to 90 cycles each, we recorded 4082 trajectories (i.e., *N*≈ 4000 larvae) and identified 2409 CSS comprising a total of 17,964 swim bouts. All trajectories that lasted more than 10 s showed a CSS. In order to tune the sensory information available to the larvae, half of them were chemically treated with CuSO_4_ to inactivate the lateral line (see Materials and Methods) and, independently, half the experiments were performed in the dark, thus leading to four different sensory conditions.

### 2.2. Transition from exploration to counterflow swim

In the absence of visual and hydrodynamic cues, the larvae passively drifted toward the suction point (Figure 2B and Supplementary Video 2). We sometimes observed brusque, oversized, and non-oriented escape bouts (see Supplementary Video 2), indicating that the remaining sensory systems (tactile, proprioceptive, and vestibular systems for instance) can trigger escape responses but are unable to generate CSS behavior on their own. All sensory conditions involving either the lateral line or the visual system showed CSS (Figure 2B and Supplementary Videos 3–5).

**Figure 3:**
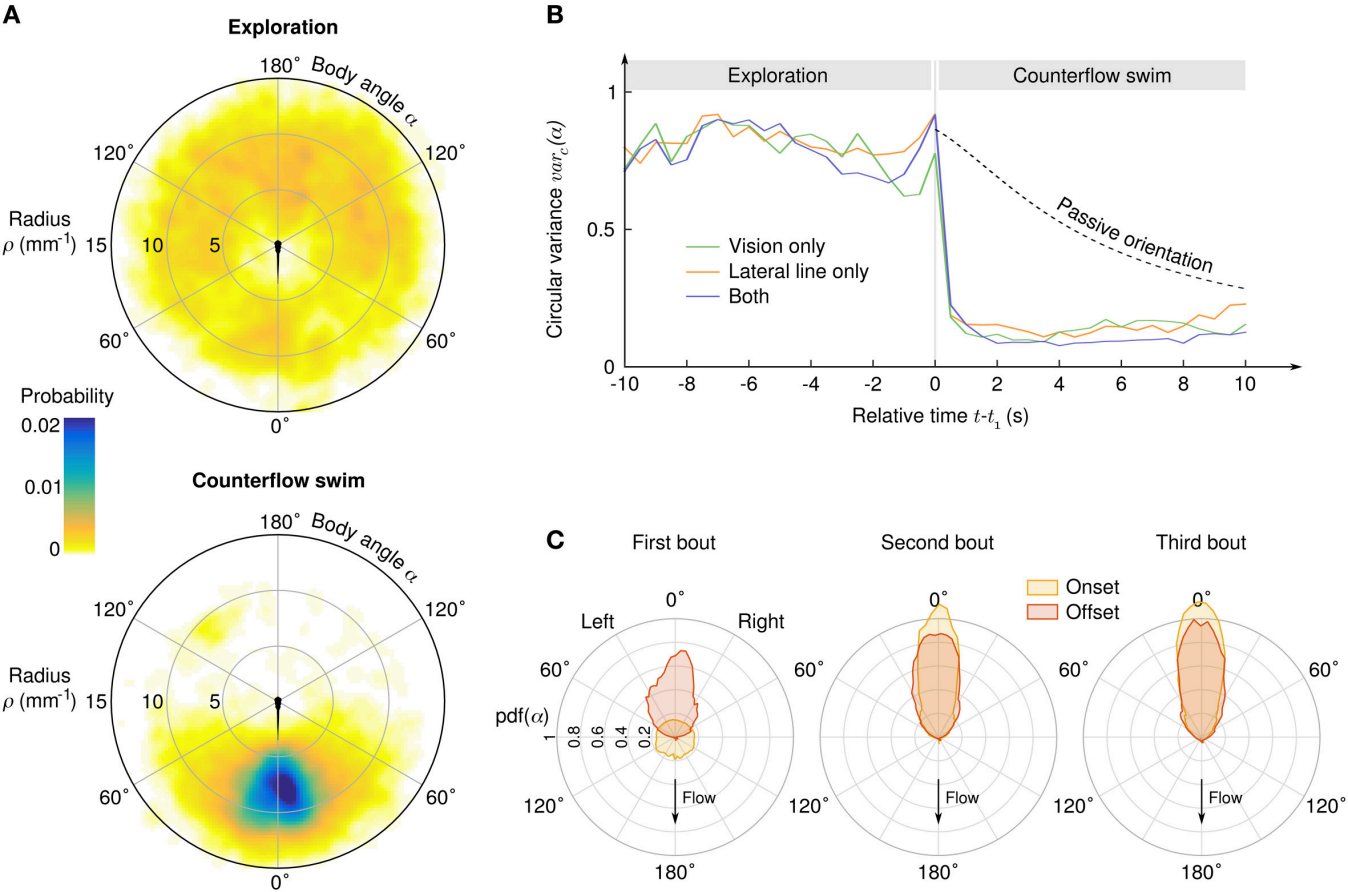
**Transition from exploration to counterflow swim**. **(A)** Probability density of the suction point's location in the reference frame of the larva, before (top) and during (bottom) the counterflow swim sequence. **(B)** Circular variance of α as a function of the time relative to the first bout of the CSS, for three different sensory conditions. The “passive orientation” curve has been obtained numerically by computing the circular variance of putative trajectories of inert larvae with the same initial conditions (position and body angle). **(C)** Distributions of the body angle α at the onset (yellow) and offset (red) of the first three bouts of the CSS.

As the angle α between the body axis and the local stream lines determines the efficiency of each impulse to compensate the flow, it is a key parameter of counterflow swim. We observed a clear transition from initial exploration behavior for which α is uniformly distributed to CSS with peaked distribution of α (Figure 3A). Aligment along the stream lines can be achieved either actively during swim bouts, or simply passively because of the *Jeffery effect*: hydrodynamic computations show that elongated objects in sheared flows experience torque (Jeffery, 1922; Dhont and Briels, 2005) which, in our geometry, tends to align the larvae radially on a time scale that depends on the flow rate and the initial position (radius and angle) of the larva. We computed the circular variance of α in time bins of 0.5 s ranging from 10 s before to 10 s after the onset of the CSS (Figure 3B), and compared it with the circular variance of simulated passive trajectories (see Materials and Methods). During the exploration phase the circular variance was close to 1, the theoretical value for a uniform angular distribution. Notably, the circular variance did not decreased with time, suggesting that the larvae actively explored their angular space at a pace that is sufficient to neutralize the effect of passive orientation (Supplementary Videos 6, 7). During the CSS the circular variance immediatly dropped to an asymptotical value close to 0, the theoretical value for perfect alignment. Quasi-radial orientation was achieved in one single bout for the three sensory conditions. For all later bouts, the distributions of body angles at the bout onset were slightly more peaked than at the bout offset, most likely due to the passive orientation occuring during inter-bout delays (Figure 3C).

### 2.3. Initiation and regulation of counterflow swimming sequences

**Figure 4:**
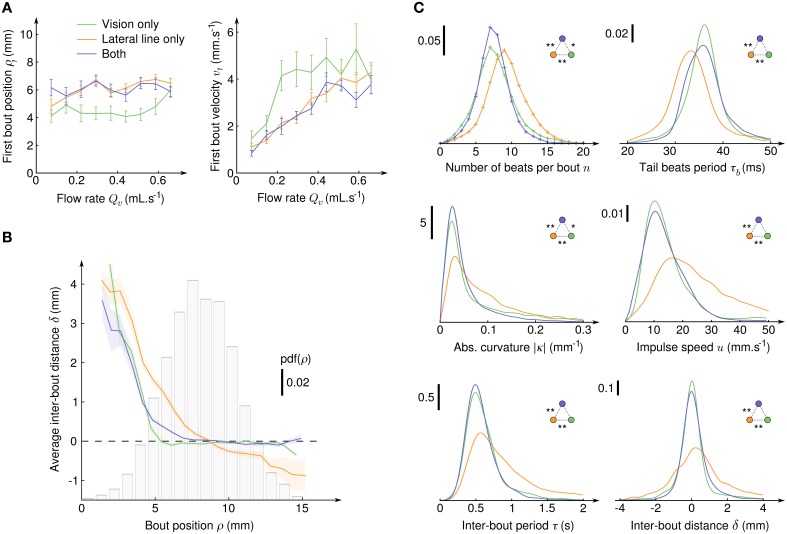
**Initiation, characterization and regulation of counterflow swim sequences**. **(A)** Average radial position ρ_1_ (left) and fluid velocity *v*_1_ (right) at the onset of the first bout of the CSS as a function of the suction flow rate, for the three sensory conditions where CSS is observed. Error bars: standard error. **(B)** Average inter-bout distance δ as a function of the bout radial position ρ during the CSS. The dashed line at δ = 0 indicates perfect distance holding. The bar plot shows the probability density function of ρ for all three sensory conditions. The colored transparent surfaces indicate standard errors. **(C)** Probability density functions of six quantities characterizing the intra- and inter-bout behavior during the CSS for the three sensory conditions. Pdf were obtained with kernel density estimation with Gaussian kernels: $\sigma_{k}^{n}=0.75$, $\sigma_{k}^{\tau_{b}}=1$, $\sigma_{k}^{\left|\kappa \right|}=0.01$, $\sigma_{k}^{u}=2$, $\sigma_{k}^{\tau }=0.075$, and $\sigma_{k}^{\delta }=0.25$. Two-sample Kolmogorov-Smirnov tests were used to determine if distributions are significantly different from each other (^*^p < 10^−3^ and ^**^p < 10^−6^).

To evaluate which hydomechanical cues are determinant in the initiation of CSS in larval zebrafish, we compared the average radial position ρ_1_ and average velocity *v*_1_ at the onset of CSS as functions of the suction flow rate (Figure 4A). For all sensory condition, the radial position was found to be independent of the flow rate, the velocity had a linear dependence and the acceleration a parabolic dependence (not shown). We checked that the distributions of ρ_1_ are different from random positions in the field of view (two-sample Kolmogorov-Smirnov test, *p* < 10^−10^ in all three sensory conditions), while the distributions of angular positions θ_1_ could not be significantly differentiated from uniform randomness (two-sample Kolmogorov-Smirnov test, *p* > 10^−3^ in all three sensory conditions). Notably, the average ρ_1_ were significatively lower when the fish relied on its sole visual system than when it could use its lateral line (Welch's *t*-test, *p* > 0.9 for lateral line only compared to both sensory input, *p* < 10^−10^ otherwise) and, coherently, the average *v*_1_ were higher. In our paradigm where the fish is driven toward the suction point it means that the lateral line allows for an earlier initiation of the CSS behavior than the visual system. With both sensory modalities, the radii at which CSS initiates are undistinguishable from those of the lateral line only condition, so the lateral line can be considered as the dominant sensory system.

After CSS initiation, the distributions of difference in angular position θ between the bouts' onsets and offsets were found Gaussian with large standard deviations (15.4° with lateral line only, 18.6° with vision only and 8.4° with both), indicating that in the course of a trajectory the larvae orientation in the laboratory frame θ varies significantly, although its relative position with respect to the suction point (ρ,α) is held (Figure 3A). Though the regulation of the body angle α during CSS appeared to be independent of the sensory condition, the regulation of radial position ρ was found to rely on different mechanisms (Figure 4B). With the lateral line only, the average inter-bout distance δ was positive close to the suction point (repulsion) and negative far away (attraction), leading to a stabilization process around an operating point corresponding in the assay to a circle of radius 8.7 mm around the suction point. With vision only, steep repulsion occured below a threshold at 5 mm, while above the threshold a plateau at δ = 0 indicated efficient radial position holding at any distance from the suction point. With both sensory modalities, the fish behaves as with the sole visual system.

The inter-bout distance δ is the result of a complex process involving the inter-bout period τ tuned by the fish and the coupling between body movements and the surrounding medium resulting in bout impulses. The impulse amplitude reads *m*.*u* where *m* is the mass of the larva and *u* is the impulse speed and depends on the number *n* of tail beats, the beating period τ_*b*_ and tail curvature κ. The distributions of *n*, τ_*b*_, |κ|, *u*, τ, and δ are displayed on Figure 4C for the three sensory conditions, as well as the outcome of pairwise two-sample Kolmogorov-Smirnov tests. It appeared that for all these quantities the distributions for the lateral line only and for the visual system only were significantly different, and that the distributions for fishes having both modalities were either very close or undistinguishable from the distributions of the visual system only condition. The visual system thus appeared to be clearly dominant in controlling the swimming patterns during CSS.

### 2.4. Maturation of the lateral line and visual systems

**Figure 5:**
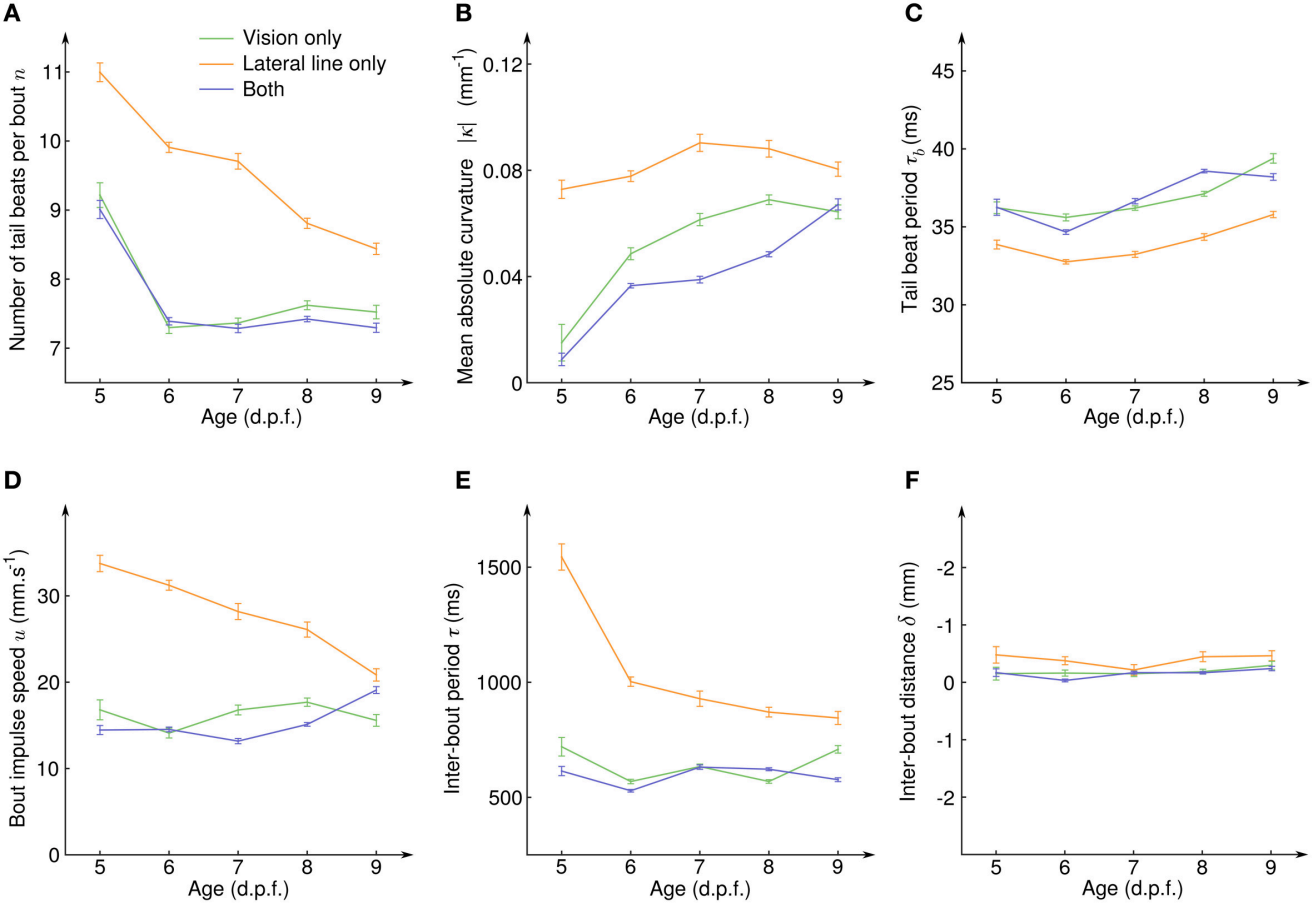
**Evolution of the counterflow swimming patterns with age for the different sensory conditions**. **(A)** Average number of tail beats per bout *n*, **(B)** mean absolute curvature |κ|, **(C)** mean tail beat period τ_*b*_, **(D)** average bout impulse speed *u*, **(E)** average inter-bout period τ, and **(F)** average inter-bout distance δ as a function of the larval stage for the three sensory conditions showing CSS. Error bars: standard errors.

To quantify how the CSS evolved with the development of the sensory systems and the associated neural circuits, we exploded the same data according to the age of the larvae (Figure 5). For all sensory modalities and at all age, the average inter-bout distance δ was close to zero (Figure 5F), indicating than an efficient position holding was always performed. However, how this net result was achieved evolved with age in a different manner for the lateral line and the visual system. With the lateral line system only, the number of tail beats per bout *n* decreased with age (Figure 5A) while the mean absolute tail curvature |κ| and the tail beat period τ_*b*_ remained constant (Figures 5B,C). This resulted in a decreasing bout impulse speed *u* (Figure 5D) which was compensated by a higher swim pace, i.e., a decrease of the inter-bout period τ (Figure 5E). With the visual system only, the number of tail beats per bout also decreased (Figure 5A) but it was directly compensated by an increase of the mean absolute curvature |κ| (Figure 5B) and all other quantities (tail beat period, impulse speed, and inter-bout period) remained constant with age. When the fish had both sensory inputs, all the measured quantities showed similar average values than for the visual system only.

## 3. Discussion

We have developed an assay for studying rheotaxis of freely swimming zebrafish larvae in radial flow geometry. The trajectories revealed two distinct swim phases: exploration, characterized by an active randomization of orientation, and CSS, characterized by an active locking onto the local streamlines direction. The transition between the two swim phases was characterized by a clear and instantaneous reorientation for all sensory conditions showing CSS and at all ages. Passive reorientation was found to be a minor effect in both phases due to the rapid pace of active bouts. During the CSS, the hydromechanical stabilization around α = 0 nevertheless facilitated orientation locking by balancing the minute swerves of counterflow swim bouts.

We found that the CSS are triggered on average at a fixed radial distance from the suction point, independantly of the flow rate. This is an exquisitely relevant feature for survival, since it ensures predator avoidance at any flow, even minute. However, the precise combination of sensory cues that allow this behavior is still to be elucidated. Many candidates can be ruled out, including for instance thresholding over any instantaneous cue (e.g., fluid speed, acceleration, hydromechanical gradients, shear rate, etc.) as they depend linearly or super-linearly on the flow rate. It is therefore likely that CSS are initiated on the basis of temporal integration of a quantity that is accessible by both the visual system and the lateral line, but the frequent bouts performed during the exploration phase makes the extraction of a precise quantity very difficult.

Regarding position holding during CSS, two different mechanisms have been found. Though the net result is the same—the fish maintains its position on average in all conditions and at all ages—the compensation mechanisms are different. On one hand, with the visual system position holding is allowed at any distance from the suction point provided it is further than a repulsion limit at ≈1.5 body lengths, and is adjusted during larval development at the level of the bout itself by the number of tail beats and the tail curvature. On another hand, lateral line-mediated position holding relies on attraction and repulsion around an operating point and is tuned during larval development by both the average number of tail beats per bout and the inter-bout period.

The lateral line and visual systems proved to be dominant for distinct tasks. The lateral line, which offers earlier flow recognition, controlled the CSS initation radial position. After CSS initiation, the lateral line system was superseded by the visual system in all aspects of the swimming patterns, down to the tail beat scale. If the larva integrated inputs from both sensory modalities to build its behavioral response, we would expect the latter to differ from the single-modality behaviors and to observe e.g., a mixture of the behavioral features. However, for both CSS triggering and position holding none of measured quantities showed a clear difference with a single-modality condition; by contraposition, we deduce that the absence of intermediary behavior rules out the possibility of multi-sensory integration during rheotaxis in this age range, in favor of multi-sensory prioritization. In addition, the fact that the swim paterns during CSS are controlled by the visual system does not mean that the lateral line system does not play a role in position holding, and, coherently with the litterature, our results demontrate that it is indeed used as a fallback mechanism. This is ethologically relevant to ensure survival during night time, in opaque water or when the visual system is damaged. Similarly, without the lateral line system the visual system can still trigger CSS, which might be useful in other flow geometries (e.g., with weak velocity gradients).

A closely related behavior is the escape response of small aquatic animals to predators using suction feeding (Engeszer et al., 2007), as the animal has swim away from the predator. The ability to detect and avoid high speed radial flows is critical for their survival, and is likely to be a multisensory process sharing many similarities with rheotaxis in milder flows. Though it is possible to simulate a predator strike in a linear flume (McHenry et al., 2009), the radial flow geometry is more realistic and allows for high experimental troughput. With our apparatus, it would be certainly of great interest to monitor and characterize the transition between position holding and escape responses as the flow rate increases.

## 4. Materials and methods

### 4.1. Fish maintenance and preparation

Zebrafish (*Danio Rerio*) embryos were obtained from natural spawning of AB wild-type lines. Larvae where reared in Petri dishes on a 14/10 h light/dark cycle at 28°. Eggs where kept in E3 solution with 10^−5^% Methylene Blue before etching, and then in standard E3 solution. Larvae where fed powdered nursery food every day from 4 to 9 days post-fertilization (dpf). In preliminary experiments we couldn't observe any CSS for larvae younger than 5 dpf, so we used larvae from 5 to 9 dpf for the assay. All experiments were carried out in accordance with approved guidelines and approved by *Le Comité d'Éthique pour l'Expérimentation Animale Charles Darwin* (02601.01).

For experiments in which the lateral line was chemically ablated, larvae were bathed in 10 μM copper sulfate (CuSO_4_, Sigma-Aldrich) during 2 h and rinsed several times in E3. All the experiments where then performed within 5 h to ensure the neuromasts did not regenerate. We checked the ablation by exposing treated larvae to 0.5 mM DASPEI solution (Sigma-Aldrich) for 40 min and observed the skin with a fluorescence binocular. All neuromast sites showed no or extremely weak fluorescence as compared to control larvae.

### 4.2. Assay

The assay consisted in a 150 mm-wide Petri dish (Sigma-Aldrich) containing 40–50 zebrafish larvae. A 0.9 mm-inner diameter needle (NN-2038S, Terumo) with a customly flattened and blunted tip was placed horizontally close to the center of the container to generate the aspiration flow (Figure 1A). A rectangular piece of transparent polymer (PMMA - 25 × 20 × 4 mm) lied in the field of view at the bottom of the Petri dish to locally reduce the water depth down to 4 mm. The suction needle was tubed to a ballast tank, a peristaltic pump (Ismatec IPC, Wertheim, Germany) and finally a reservoir tank filled with E3. The ballast smoothed the jerky flow generated by the peristaltic pump at high flow rates and served as a storage tank for larvae sucked during the experiment. Each experiment consisted in series of 90 suction/injection cycles. The flow rate during the suction phase was randomly choosen among nine different values ranging linearly from 0.07 to 0.66 mL.s^−1^. Suction phases lasted no more than 20 s, such that the water depth change was neglectable even at the highest flow rate. During the injection phase, the same amount of water was reinjected in the assay at 0.22 mL.s^−1^. An electrovalve (LHDA0533115H, The Lee Company) redirected the reinjected flow to a second syringue tip located far away from the suction point (Figure 1B). Orthoradial reinjection created a circular flow in the Petri dish which tended to gather the larvae at the center, in the field of view. A 10 s pause was marked between each reinjection and the next run to let the circular flow vanish. Injections/reinjections cycles and camera acquisition were fully automatized and managed by a custom set of programs using LabVIEW (National Instruments, Texas, USA), FlyCapture2 (Point Grey Research, Richmond, BC, Canada), and Sikuli (User Interface Design Group, MIT, Massachusetts, USA).

A Flea3 USB3 Camera (Point Grey Research, Richmond, BC, Canada) with an adjustable macro lens (Zoom 7000, Navitar, USA) recorded a 22 × 18 mm region of interest in the assay from above with a pixel size of 35 m. Trajectories were recorded by the camera in free-running mode at an average framerate of 250 frames.s^−1^ with pixel-encoded timestamps. A dark background was placed under the assay. Raking illumination of the scene was performed with two high-power infra-red LEDs (850 nm, SFH 4750, Osram). An IR filter (LS387111 LO, Goodfellow, Huntingdon, England) placed in front of the lens blocked all visible light. A 45° mirror was placed in the field of view (Figure 1A) to check that larvae where not touching the bottom of the assay. Due to focus loss the mirrored images were too blurred to allow for precise extraction of the vertical position of larvae.

### 4.3. Image processing

All data was analyzed using custom-written software in Matlab. On each image, larvae were detected by (i) substracting the run's average image, (ii) applying a Gaussian filter (175 × 175 μm box, σ = 50 μm), (iii) thresholding and filtering objects smaller than 500 pixels (~50% of a larva).

To evaluate body curvature, we used an approach based on image moments inpired by the work of Rocha et al. (2002) for tracking articulated objects. We evaluated the level-1 *BSP*-tree to obtain two equivalent ellipses of the head and the tail of each larva at each time frame (Figure 1C and Supplementary Video 1). The body angle α was defined as the angle between the major axis of the head ellipse and the radial direction. We then considered the intersection of both ellipses' minor axes as the center of curvature and computed the radius of curvature *R* as the average distance between the center of curvature and all larva's pixels. The curvature was defined as κ = 1∕*R* and signed positively when the center of curvature was located on the right of the larva, negatively otherwise. This quantity accurately described the swim tail beats, even at very low amplitude (Supplementary Video 1).

The larvae were then tracked among all images with the algorithm described in Crocker and Grier (1996) with a maximal dispersion of 3.5 mm between two images and a memory of 100 time steps (400 ms). We used a custom graphical user interface to visualize the radial and angular trajectories, bouts and fits (described in the Data Analysis paragraph). We used this interface to manually discard the trajectories that are too short, for which the larva sticked to the bottom of the assay or multiple larvae contacted each other. Series of bouts with a clear motion away from the suction source were defined as CSS.

### 4.4. Data analysis

All data was analyzed using software custom-written in Matlab.

Swim bouts were automatially detected based one the curvature signal κ. We first computed $\sigma_{\dot{\kappa }}$ the standard deviation of the derivative of the curvature over a sliding window of 20 ms, and divided it by a baseline trace obtained by taking the average of $\sigma_{\dot{\kappa }}$ below the median value over a large sliding window of 400 ms. Swim bouts were localized when the normalized $\sigma_{\dot{\kappa }}$ stood above a threshold of 5 for more than 40 ms. In each swim bouts the tail beats were automatically detected when the absolute relative curvature stood above a threshold of 0.1.

For each bout, the time at which the radius ρ(*t*) was minimal defined the impulse start *t*_*k*_. The radial position traces were then fitted on the 100 ms preceeding each bout by an affine function ρ(*t* < *t*_*k*_) = *v*_*f*_*t* + *c* to extract the fluid velocity *v*_*f*_, and on the 300 ms following each bout (or less if there was another bout in this range) by:

(1) $$\rho (t>t_{k})=\rho (t_{k})+\lambda ucos(\alpha )(1-e^{-t/\lambda })-v_{f}t$$

where *u* is the impulse velocity and λ is the Stoke's drag damping time scale. The latter can be written λ = Γμ∕*m* where μ is the dynamic viscosity, *m* is the mass of the larva and Γ is a constant solely depending on the geometry of the larva. The typical dimensions and mass of the larvae changed from 5 to 9 dpf (Supplementary Figure 1A), but this did not affected the distribution of the fitted λ (Kolmogorov-Smirnov test, *p* = 10^−2^ between 5 and 9 dpf - Supplementary Figure 1B).

The circular variance of body angles α_*k*_ at the bouts' onsets is defined as:

(2) $$var_{c}(\alpha )=1-|\frac{1}{N}\sum_{k=1}^{N}e^{i\alpha_{k}}|$$

and varies from 0 for minimal dispersion (all angles are similar) to 1 for maximal dispersion (angles compensate exactly, or the distribution is uniform and *N* is large).

### 4.5. Simulation of passive reorientation

In our geometry the angular speed of a passive larva can be approximated by the non-linear first-order differential equation:

(3) $$\dot{\alpha }=\frac{q}{L\rho }sin(\alpha )$$

with *L* the length of the larva, and $q=\frac{Q_{v}}{2\pi h}$ the surfacic flow rate. This equation holds when ρ≫*L*, and was solved numerically. We took as initial condition the actual position and body angle of the larvae at the beginning of each experiment.

## Author contributions

RO designed and realized the experimental setup, performed the experiments, and contributed to image processing and data analysis. SW contributed to image analysis and animal tracking. AD and VB contributed to data analysis. GD contributed to data analysis and article writing. RC designed the experiment, analyzed data, and wrote the article.

### Conflict of interest statement

The authors declare that the research was conducted in the absence of any commercial or financial relationships that could be construed as a potential conflict of interest.

## Supplementary material

Supplementary Figure 1 **Evolution of the drag coefficient during larval development. (A)** Typical dimensions of zebrafish larvae at 5 and 9 dpf. **(B)** Probability density function of the drag coefficient λ at different larval stages.
