# Supplementary figures and images for: Rheotaxis of Larval Zebrafish: Behavioral Study of a Multi-Sensory Process
Source: Front Syst Neurosci. 2016 Feb 23;10:14. doi: 10.3389/fnsys.2016.00014 (PMC4763089; doi:10.3389/fnsys.2016.00014)

**A**

5 dpf

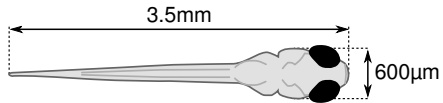

9 dpf

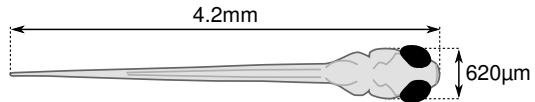**B**

Probability density function

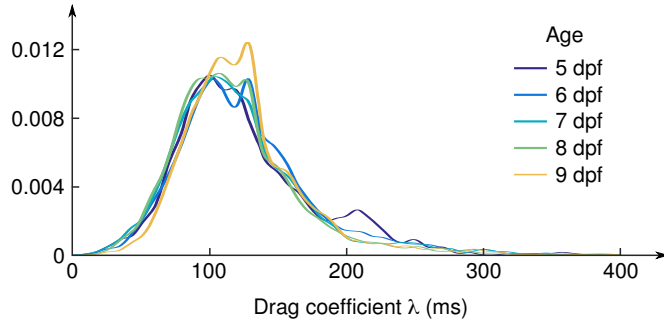

Supplement: Supplementary Figure 1 — Evolution of the drag coefficient during larval development. (A) Typical dimensions of zebrafish larvae at 5 and 9 dpf. (B) Probability density function of the drag coefficient λ at different larval stages. [file SupplementaryFigure1.pdf]
